# Stiffness and Tissue Viscosity in a Cerebral Neoplasm Measured by Preoperative Multifrequency Magnetic Resonance Elastography (MRE) Guide the Differential Diagnosis of Brain Tumors by Ruling Out Glioma

**DOI:** 10.1155/carm/3863128

**Published:** 2026-06-24

**Authors:** Mehrgan Shahryari, Boshra Shams, Helge Herthum, Pablo Gottheil, Jeremias Heider, Josef Käs, Eberhard Siebert, Thomas Picht, Ingolf Sack

**Affiliations:** ^1^ Department of Radiology, Charité-Universitätsmedizin Berlin, Corporate Member of Freie Universität Berlin and Humboldt-Universität zu Berlin, Berlin, Germany; ^2^ Berlin Institute of Health at Charité-Universitätsmedizin Berlin, BIH Charité Junior Digital Clinician Scientist Program, BIH Biomedical Innovation Academy, Charitéplatz 1, Berlin, 10117, Germany; ^3^ Department of Neurosurgery, Charité-Universitätsmedizin Berlin, Corporate Member of Freie Universität Berlin and Humboldt-Universität zu Berlin, Berlin, Germany, charite.de; ^4^ Berlin Center for Advanced Neuroimaging, Charité-Universitätsmedizin Berlin, Corporate Member of Freie Universität Berlin, Humboldt-Universität zu Berlin, and Berlin Institute of Health, Berlin, Germany; ^5^ Faculty of Physics and Earth Sciences, Peter Debye Institute, Leipzig University, Leipzig, Germany, uni-leipzig.de; ^6^ Department of Neuroradiology, Charité-Universitätsmedizin Berlin, Corporate Member of Freie Universität Berlin and Humboldt-Universität zu Berlin, Berlin, Germany

## Abstract

Here, we report the case of a 65‐year‐old man who presented to the emergency department with paresthesia, psychomotor retardation, and dysarthria. Contrast‐enhanced brain MRI revealed a solitary, contrast‐enhancing mass in the right head of the caudate nucleus with extensive perifocal edema, raising a primary differential diagnosis of glioblastoma versus metastasis, among other possibilities. Prior to surgical removal of the suspected glioblastoma, the patient underwent additional diffusion tensor imaging and research MRI, including multifrequency magnetic resonance elastography (MRE) to generate mechanical property maps of stiffness and tissue viscosity and to potentially differentiate between glioma, brain metastases, and benign masses based on tissue biomechanical properties. MRE revealed abnormally high values of stiffness and viscosity—a mechanical signature known to be associated with brain metastases or meningioma, but not glioblastoma, which typically exhibits soft tissue properties with reduced viscous dissipation. Histopathologic evaluation confirmed MRE by revealing a distant metastasis of squamous cell carcinoma, the primary tumor of which was detected by PET‐CT within the right hilus of the lung. This case demonstrates the diagnostic value of the biomechanical properties of brain tumors and raises the prospect of applying MRE as an easy‐to‐use MRI contrast that can rule out glioma when the suspicious lesion exhibits stiff‐viscous material properties.

## 1. Introduction

Individuals with relatively fast‐growing brain tumors such as metastases or glioblastomas typically present with headaches, nausea, vomiting to their general practitioner, or, in cases, involving seizures or focal neurological deficits to the emergency department [1]. The diagnosis of brain tumors is then made by imaging studies based on CT and MRI [1, 2]. Contrast‐enhanced MRI is the recommended diagnostic modality for clinical suspicion of brain tumors [3]. However, differential diagnosis and histopathologic confirmation require invasive biopsies or tissue sampling after resection [4]. Additional information obtained noninvasively through imaging studies is highly desired to further improve the clinical diagnosis of brain tumors in a wide range of neurological tumor entities.

A fundamental property of brain tumors with implications for surgical resection is viscoelasticity [5]. Surgeons sense the mechanical consistency of brain tumors by their resistance to deformation [6]. Effective resection of brain tumors and detection of tumor boundaries often depend on the haptic discrimination between tumor and adjacent tissue. Magnetic resonance elastography (MRE) can visualize the viscoelastic properties of the brain, including soft‐matter lesions [7–9]. In recent years, multifrequency MRE and tomoelastography have matured into a fast, robust MRI technique for characterizing healthy and tumorous brain tissue according to their mechanical properties [10]. In the literature, various studies have shown that MRE can aid in the differentiation of benign from malignant masses and their imaging‐based characterization [11, 12]. Recent studies have shown that gliomas exhibit heterogeneous, soft mechanical properties with decreased viscosity‐related parameters compared to contralateral normal‐appearing white matter [9, 13–17]. In particular, experimental multifrequency MRE studies have reported that gliomas exhibit lower viscous dissipation compared to meningiomas, making the tissue loss angle a potentially valuable imaging marker for their differential diagnosis [18].

More recently, various advancements in MRI techniques have been explored to distinguish gliomas from brain metastases, including proton MR spectroscopy (1H‐MRS), diffusion‐weighted imaging (DWI), and perfusion MRI [17, 19–23]. However, conventional MRI often presents challenges in differentiating solitary brain metastases from gliomas based on radiological assessments, particularly when patients exhibit a single lesion with an enhancing rim and peritumoral edema. Therefore, high‐grade gliomas could be misinterpreted as solitary brain metastases and vice versa [24, 25].

Here, we report the use of novel tomoelastography based on multifrequency MRE of the brain in a patient with suspected glioblastoma. Tomoelastography provided maps of shear wave speed (SWS), penetration rate (PR), and loss angle (*ϕ*) as proxies for tissue stiffness, penetration, and viscous dissipation [26]. The obtained mechanical profile clearly indicated that the suspicious mass was not a glioma, despite typical signs of a glioblastoma in contrast‐enhanced MRI. Consequently, the further treatment plan was initially outlined based on guideline‐approved MRI including surgical resection [27, 28].

## 2. Case Presentation

A 65‐year‐old man with no family history of malignant disease presented to his general practitioner with progressive paresthesia of the upper extremity, psychomotor retardation, and dysarthria for several weeks. Additionally, the patient reported that his left leg seemed to lag during prolonged walking. He was apparently well 2 months prior. There were no associated symptoms of nausea, vomiting, seizure, or dizziness. The patient had no B‐symptoms and no previous illnesses and did not take any medication. He reported no known allergies and a smoking history of 20 pack‐years.

The patient was referred for an outpatient MRI (Figure 1). The outpatient MRI consisted of T2‐weighted FLAIR, T2‐weighted transversal MRI, diffusion‐weighted MRI for quantification of apparent diffusion coefficient (ADC), 3‐dimensional (3D) time‐of‐flight (TOF) contrast‐enhanced MRI, T1‐weighted, contrast‐enhanced transversal and coronal MRI, sagittal MPRAGE, and susceptibility weighted imaging (SWI) with transversal slice orientations. Outpatient MRI demonstrated an irregular lesion in the head of the caudate nucleus, with predominantly peripheral contrast enhancement surrounding a T2‐hyperintense central necrotic core (Figure 1). The tumor periphery showed solid components with diffusion restricted areas and low intertumoral susceptibility signals due to microhemorrhage and vascular proliferation. A large edema surrounded the brain tumor.

**FIGURE 1:**
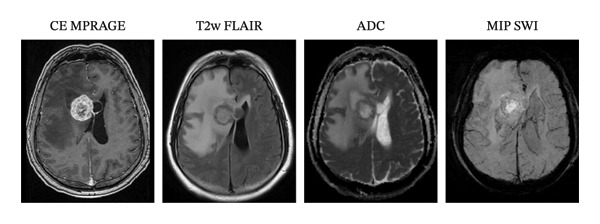
Ex domo MRI 2 days before surgery contrast‐enhanced. MPRAGE, T2‐weighted FLAIR, ADC, and SWI images from the outpatient MRI reveal a solitary brain mass in the right frontal lobe with predominantly peripheral contrast enhancement and a T2‐hyperintense central necrotic core adjacent to the right anterior horn of the lateral ventricle. The tumor periphery exhibited solid components with diffusion‐restricted areas, low intertumoral susceptibility signals suggestive of microhemorrhage, and evidence of vascular proliferation. A large T2‐hyperintense peritumoral edema and a small midline shift towards the left were also observed.

On the same day, the patient presented to our emergency department with the external MRI, which raised suspicion of glioblastoma. Therefore, he was admitted to the neurosurgical ward for further evaluation and treatment.

One day prior to surgical resection of the tumor, the patient underwent a clinical MRI examination (3‐Tesla Magnetom Lumina, Siemens Healthineers, Erlangen) including diffusion tensor imaging (DTI), MPRAGE, 3D T2‐weighted, 3D T2‐weighted FLAIR sequences for anatomical reference, as well as multifrequency MRE.

Multifrequency MRE was performed using four sequential mechanical vibration frequencies (20, 25, 30, and 35 Hz), delivered by two pressurized air drivers (THEA devices, Wurzen, Germany) positioned at the occiput [26]. The drivers were securely fixed using a custom‐made placeholder, specifically adapted for the air drivers and head coil configuration (Figure 2A) [26]. The drivers were activated in an opposed‐phase configuration to minimize anterior–posterior bulk motion artifacts. A 3‐s frequency forerun preceded each acquisition to establish steady‐state harmonic vibration. Eight phase offsets for each 3D wavefield were acquired from 40 transverse slices (field of view: 204 × 204 mm^2^; matrix: 102 × 102; slice thickness: 2 mm) using a single‐shot spin‐echo echo‐planar imaging (EPI) sequence with flow‐compensated motion‐encoding gradients (MEG). Further details regarding the MRE setup and sequence parameters are provided by Herthum et al. [26]. Figure 2B shows frequency‐resolved wavefields for each component. Prior to tomoelastography processing, all complex images were registered to the first image based on magnitude using the open‐source elastix toolbox for motion correction, as described by Shahryari et al. [29]. The processing pipeline implemented a brain‐adapted wavenumber‐based multifrequency dual elasto‐visco (k‐MDEV) inversion method, as detailed by Herthum et al. [26] and accessible via https://bioqic-apps.charite.de [30]. In essence, following temporal Fourier transformation, the complex displacement fields were decomposed into eight shear wave components and band‐pass filtered (high‐pass threshold, 15 m^−1^; low‐pass threshold, 300 m^−1^) to suppress low‐frequency compression waves and noise. Local complex wavenumbers were derived from spatial phase gradients at each frequency. Frequency‐resolved maps of SWS (m/s; stiffness), PR (m/s; penetration), and loss angle (*φ*, rad, viscous dissipation) were generated and averaged across mechanical frequencies. Displacement SNR, used to ensure sufficient wave amplitude, was 30 dB and 27 dB for the cases in Figure 3 and 4, respectively [31, 32]. The loss angle *φ* quantifies a material’s tendency toward viscous deformation, as reflected by the complex shear modulus (i.e., a larger loss modulus relative to the storage modulus); higher loss angle (*φ*) values indicate more pronounced dissipative behavior. This angle ranges from 0 (purely elastic behavior) to *π*/2 (purely viscous behavior) [10, 18]. Regions of interest (ROIs) encompassing the contrast‐enhancing tumor and contralateral normal‐appearing white matter were manually delineated on coregistered contrast‐enhanced T1‐weighted images and applied to the mechanical parameter maps (Figure 3 and 4).

**FIGURE 2:**
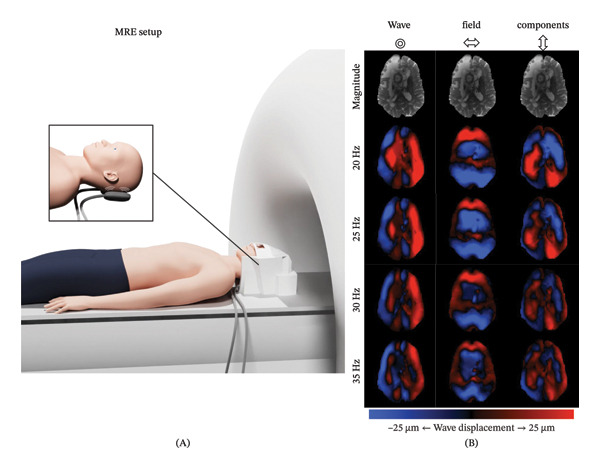
MRE setup. (A) Multifrequency MRE vibrations were induced by two 3D‐printed air cushion actuators positioned within the head coil beneath the patient’s head. The actuators were driven by pressurized air pulses at frequencies ranging from 20 to 35 Hz and delivered through connecting tubes. (B) Wave fields for each component and frequency (20, 25, 30, and 35 Hz), as well as corresponding frequency‐averaged MRE magnitude images for each component. The blue‐to‐red color scale represents displacement amplitudes of 25 μm.

**FIGURE 3:**
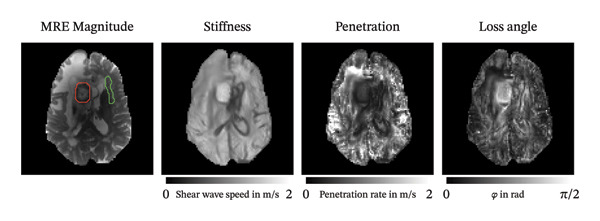
Preoperative MRE one day before surgery. MRE magnitude, stiffness (shear wave speed, SWS), penetration (penetration rate, PR), and loss angle (*φ*) maps of a patient with a right frontal brain tumor. MRE depicts the tumor (red contour) as a homogeneous, stiff lesion with reduced penetration and increased loss angle relative to contralateral normal‐appearing white matter (green contour). Mean shear wave speed, penetration rate, and loss angle in tumor versus normal‐appearing white matter were 1.43 ± 0.17 m/s versus 1.01 ± 0.08 m/s, 0.4 ± 0.09 m/s versus 0.79 ± 0.29 m/s, and 1.06 ± 0.16 rad versus 0.48 ± 0.16 rad, respectively.

**FIGURE 4:**
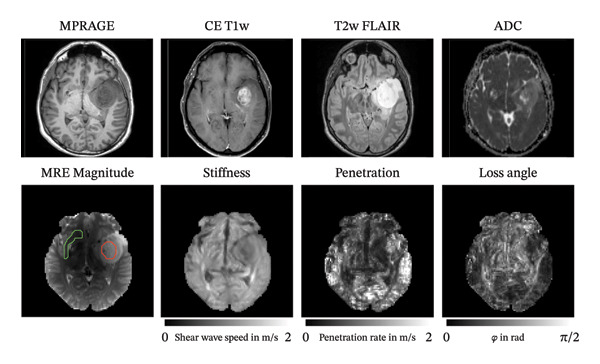
Comparison case of a WHO grade IV glioma. MPRAGE, contrast‐enhanced T1‐weighted imaging, T2‐weighted FLAIR, and ADC images of a histopathologically diagnosed WHO grade IV glioma in the left temporal lobe, showing predominantly peripheral contrast enhancement. Similar to the case shown in Figure 3, the glioma periphery exhibited solid components with diffusion restriction and elevated ADC values in the central necrotic region. T2‐hyperintense peritumoral edema and a minimal midline shift to the right were also observed. Tomoelastography depicts the tumor (red contour) as a heterogeneous, soft lesion with increased penetration and decreased loss angle relative to contralateral normal‐appearing white matter (green region of interest). Mean shear wave speed, penetration rate, and loss angle in tumor versus normal‐appearing white matter were 0.86 ± 0.09 m/s versus 1.18 ± 0.11 m/s, 0.78 ± 0.35 m/s versus 0.82 ± 0.3 m/s, and 0.41 ± 0.17 rad versus 0.5 ± 0.15 rad, respectively.

In addition to the clinical MRI findings, the tumor demonstrated abnormally high stiffness and loss angle values (Figure 3). These mechanical characteristics are typically associated with metastases or meningiomas rather than glioblastomas, which generally exhibit soft material properties [18]. Consequently, the MRE findings were more suggestive of a brain metastasis than a glioblastoma in the differential diagnosis. Gliomas generally exhibit reduced stiffness and viscosity‐related parameters (low loss angle and high penetration) compared to surrounding and contralateral normal‐appearing white matter [9, 13–16, 18]. Figure 4 illustrates a comparison case of a histologically confirmed glioblastoma in the left temporal lobe in a 52‐year‐old man investigated using tomoelastography.

The patient underwent microsurgical tumor resection via an interhemispheric, transcallosal frontal craniotomy. The tumor appeared as a firm lesion with a capsule, allowing enucleation from the center, resulting in complete resection. Two tissue samples were sent to the neuropathology department for histological and molecular analysis. Hematoxylin and eosin (H&E)‐stained sections revealed a highly cellular, pleomorphic epithelial tumor with squamous morphology. Immunohistochemistry for glial fibrillary acidic protein (GFAP) showed no immunoreactivity in the tumor cells. Based on these findings, the histological diagnosis was consistent with a metastasis of a squamous cell carcinoma.

Postoperatively, the patient was transferred back to the neurosurgical ward after a brief stay in the intensive care unit. At no point did the patient exhibit signs of new neurological deficits. The patient demonstrated significant improvement in mobility after a short recovery period, utilizing a walker for support. The first postoperative MRI revealed an uncomplicated postoperative status, except for a minor ischemic rim along the resection margin (Figure 5). The patient was discharged from the neurosurgery ward 1 week after the surgical resection.

**FIGURE 5:**
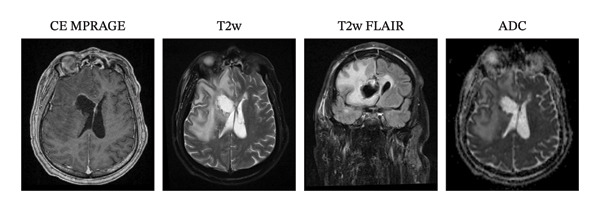
Postoperative MRI one day after surgery. Postoperative MRI obtained one day after surgery demonstrates a cerebrospinal fluid‐filled resection cavity in the right frontal lobe in the area of the former tumor, with no evidence of recurrence. A narrow diffusion‐restricted area at the laterorostral resection margin suggests a small ischemic zone. A large edema remains visible surrounding the resection area.

A contrast‐enhanced CT scan of the neck, thorax and abdomen were performed to identify the primary tumor. This revealed a 56‐mm pulmonary mass in the right hilar region. Subsequent PET/CT imaging showed evidence of contiguous mediastinal infiltration and mediastinal lymph node metastases (Figure 6). Together with further histopathological examinations, these results confirmed the diagnosis of a non–small cell lung carcinoma (NSCLC) with TNM classification of cT4, cN1, and pM1b. Genetic profiling did not indicate any targeted (immune) therapies.

**FIGURE 6:**
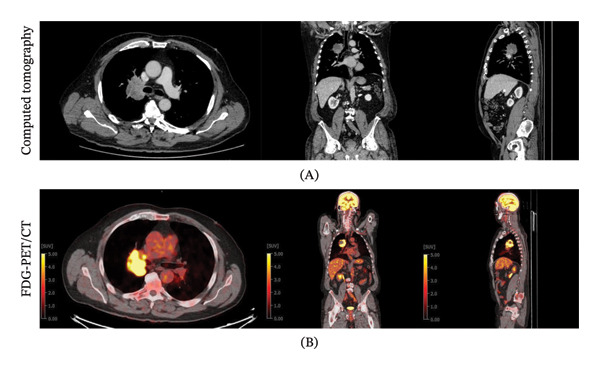
Positron emission tomography (PET) 7 days after surgery. (A) CT and (B) PET/CT scans of a patient with right frontal brain metastasis. The CT scan revealed a 56‐mm primary pulmonary mass in the right upper lobe adjacent to the right hilar region, as well as enlarged lymph nodes in the right hilum and mediastinum. PET/CT demonstrates increased metabolic activity in the lung tumor, as well as in the brain metastasis and the enlarged lymph nodes, confirming metastatic involvement. No other metastases were detected outside of the brain and lymph nodes.

Palliative therapy was initiated in collaboration with the pulmonology and radiation oncology departments, consisting of a regimen of pembrolizumab, carboplatin, and nab‐paclitaxel, along with five sessions of stereotactic radiosurgery using CyberKnife to target the cranial resection cavity. Follow‐up cranial MRI will be performed 3 months after the final CyberKnife session.

## 3. Discussion

The reported case highlights the importance of biomechanical tumor properties for noninvasive assessment of gliomas prior to surgical intervention. MRE revealed increased stiffness and viscous dissipation of the tumor compared to the contralateral normal‐appearing white matter. Based on reports in the literature, this mechanical profile is not associated with gliomas, which typically have soft‐elastic properties, i.e., low stiffness and loss angle values as found in materials such as ultrasound gel [9, 14–16, 18]. In contrast, high stiffness and loss angle values in MRE maps indicate a dense mechanical environment combined with mechanical loss due to friction, as in highly viscous malignant liver tumors such hepatocellular carcinoma or liver metastases [10]. Recent MRE literature on gliomas reports heterogeneous soft tumor properties, particularly in glioblastoma, with reduced viscosity‐related parameters (low loss angle and high penetration) similar to the presented glioblastoma comparison case in Figure 4 [9, 14–16, 18]. Interestingly, gene expression signals from stiffer regions within heterogeneous glioblastomas have been associated with shorter patient survival [13]. In particular, the low loss angle characteristics of gliomas tend to differ from other brain tumors such as meningiomas, which exhibit higher viscosity‐related parameters but can present as either stiff or soft masses depending on their histological subtype [18, 33–37]. To date, no definitive trend has emerged for the mechanical characteristics of brain metastases [38]. This presented case delineates the brain metastasis as a stiff tumor with increased viscosity‐related parameters, underscoring the potential of presurgical noninvasive mechanical characterization of brain tumors by MRE. Knowledge of such fundamental material properties in brain tumors obtained from noninvasive MRI scans could aid in therapy planning and improve the effectiveness of surgical interventions [17, 34, 36].

There is increasing evidence that the viscoelastic properties of tumors and their surrounding tissues play a critical role in promoting proliferation and metastasis [39]. Growth in a dense environment generates mechanical stress either through displacement or infiltration of normal tissue, that is, shear and volumetric deformation, respectively [39]. Therefore, from a biomechanical perspective, solid tumors nested in nonexpandable conditions such as the brain must generate force to further proliferate and expand [39]. At the same time, cancer cells are characterized by a high degree of motility to enable collective cell behavior, invasion, and spread [40]. These two opposing principles, mechanical strength and flexibility, combined in biological soft matter, evoke unique material properties related to altered stiffness and tissue viscosity [41]. Therefore, MRE in brain tumors may provide a contrast directly linked to the in vivo biophysical cues that guide cancer cells [39, 42].

Multifrequency MRE with tomoelastography processing has emerged as a robust clinical imaging modality that can be easily integrated into standard MRI protocols [10]. In our current implementation with four mechanical vibration frequencies, the total acquisition time was 8 min. Further shortening is possible by reducing the number of slices and frequencies. The external vibrations in the chosen frequency range between 20 and 35 Hz have not been reported to be distressing for patients. In addition, MRE does not require the administration of contrast agents, making it particularly safe for patients with contrast media hypersensitivity or renal impairment.

While this case demonstrates promising clinical applicability, it is constrained by its single‐observation design, comprising only one case of brain metastasis and one glioblastoma comparison case. Such a limited sample size inherently precludes establishing diagnostic validity, reliable quantitative cutoff values, or generalizability, particularly in light of the well‐documented intra‐ and intertumoral biomechanical variability observed across brain lesions in prior MRE studies [13–16, 18]. Moreover, heterogeneous acquisition protocols, inversion algorithms, and postprocessing pipelines across different MRE methods further limit comparability with existing literature. Consequently, larger prospective multicenter studies incorporating diverse tumor histologies, matched controls, and validated standardization methods are essential to rigorously assess MRE’s potential clinical utility for noninvasive tumor differentiation.

## 4. Conclusion

This case report highlights the potential of MRE as a noninvasive imaging modality for differentiating glioblastoma from metastatic brain tumors. In contrast to glioblastoma, the metastatic lesion in this case exhibited increased stiffness and loss angle values, supporting the hypothesis that biomechanical properties can serve as discriminative biomarkers in oncological imaging. Because MRE can be seamlessly integrated into routine MRI protocols without additional risk to the patient, it may represent a valuable adjunct to conventional neuroimaging.

## Funding

This study was funded by the Deutsche Forschungsgemeinschaft, CRC1540 460333672, FOR5628 513752256, GRK2260 BIOQIC, CRC1340. Open Access funding enabled and organized by Projekt DEAL.

## Ethics Statement

The work was conducted in accordance with the ethical principles outlined in the Declaration of Helsinki. Approval was granted by the institutional review board of Charité ‐ Universitätsmedizin Berlin, and written informed consent was obtained from patients prior to participation.

## Conflicts of Interest

Ingolf Sack is co‐founder of THEA‐devices, Wurzen, Germany.

## Data Availability

Deidentified data supporting the findings of this study are available from the corresponding author upon reasonable request.
